# Predictive modeling of colorectal cancer using exhaustive analysis of microbiome information layers available from public metagenomic data

**DOI:** 10.3389/fmicb.2024.1426407

**Published:** 2024-08-26

**Authors:** Boštjan Murovec, Leon Deutsch, Blaž Stres

**Affiliations:** ^1^Faculty of Electrical Engineering, University of Ljubljana, Ljubljana, Slovenia; ^2^Department of Animal Science, Biotechnical Faculty, University of Ljubljana, Ljubljana, Slovenia; ^3^The NU, The NU B.V., Leiden, Netherlands; ^4^D13 Department of Catalysis and Chemical Reaction Engineering, National Institute of Chemistry, Ljubljana, Slovenia; ^5^Faculty of Civil and Geodetic Engineering, Institute of Sanitary Engineering, Ljubljana, Slovenia; ^6^Department of Automation, Biocybernetics and Robotics, Jožef Stefan Institute, Ljubljana, Slovenia

**Keywords:** gut microbiome, machine learning, colorectal cancer, colorectal adenoma, metagenomics, functional microbiome

## Abstract

This study aimed to compare the microbiome profiles of patients with colorectal cancer (CRC, *n* = 380) and colorectal adenomas (CRA, *n* = 110) against generally healthy participants (*n* = 2,461) from various studies. The overarching objective was to conduct a real-life experiment and develop a robust machine learning model applicable to the general population. A total of 2,951 stool samples underwent a comprehensive analysis using the in-house MetaBakery pipeline. This included various data matrices such as microbial taxonomy, functional genes, enzymatic reactions, metabolic pathways, and predicted metabolites. The study found no statistically significant difference in microbial diversity among individuals. However, distinct clusters were identified for healthy, CRC, and CRA groups through linear discriminant analysis (LDA). Machine learning analysis demonstrated consistent model performance, indicating the potential of microbiome layers (microbial taxa, functional genes, enzymatic reactions, and metabolic pathways) as prediagnostic indicators for CRC and CRA. Notable biomarkers on the taxonomy level and microbial functionality (gene families, enzymatic reactions, and metabolic pathways) associated with CRC were identified. The research presents promising avenues for practical clinical applications, with potential validation on external clinical datasets in future studies.

## 1 Introduction

The prevalence of colorectal carcinoma (CRC) as the third most common nongender-related cancer and its associated mortality after lung cancer is of great concern (Sung et al., 2021). With an aging population leading to an expected 80% increase in global incidence over the next two decades, understanding sporadic colorectal cancers has become increasingly important (Karsa et al., 2010). These non-hereditary colorectal cancers account for 70–87% of cases, with genetics accounting for only a fraction of disease incidence (Frank et al., 2017). The lack of a clear genetic link underscores the potential influence of other factors, including lifestyle and environmental components, as co-determinants of disease (Siegel et al., 2014). Certain risk factors such as age, tobacco and alcohol use, physical inactivity, increased body weight, and dietary habits have been associated with CRC, but clarification of these associations remains an ongoing challenge (Huxley et al., 2009; Johnson et al., 2013).

The human gut microbiome, which encompasses the microbial communities in the intestinal tract, is becoming increasingly important because of its role in human disease (Pasolli et al., 2016). Supported by evidence that bacterial organisms trigger carcinogenic mechanisms, the role of the gut microbiome in the development of CRC has been proposed (Wong and Yu, 2023). The association of *Fusobacterium nucleatum* with CRC was revealed by amplicon sequencing of the 16S ribosomal RNA (rRNA) gene and later confirmed as causative in animal models CRC (Kostic et al., 2012, 2013; Rubinstein et al., 2013). While 16S rRNA gene studies revealed such associations, metagenomic sequencing studies revealed a smaller number of CRC-associated microbial species and functional activities. However, the consistency and prognostic potential of these high-resolution microbial signatures across different cohorts and study designs remain uncertain. Although the use of the gut microbiome for CRC diagnostics has been proposed, its validation in multiple independent studies is still pending (Zackular et al., 2014; Zeller et al., 2014; Feng et al., 2015; Baxter et al., 2016; Yu et al., 2017).

Therefore, there remains a need to establish and validate links between the human gut microbiome and CRC across different populations, cohorts, and microbiome tools. While some cross-cohort studies have been based on 16S rRNA gene studies, this technique has its own limitations (Durazzi et al., 2021). The advent of whole-metagenome shotgun datasets for CRC cohorts facilitates a comprehensive exploration of the CRC-associated microbiome that includes strain-level precision and meta-analytic prediction strategies. Therefore, extensive cross-cohort studies are essential for an unbiased and robust assessment of the relationship between CRC and the gut microbiome.

While sequencing of gene amplicons for microbial identification, especially 16S rRNA sequencing, remains a priority, metagenomic analysis by genome-wide shotgun sequencing is becoming increasingly important. It was shown before that with shotgun sequencing entire microbial community can be screened (including viruses, fungi), especially the less abundant taxa, which can also be biologically important. On the other hand, with shotgun sequencing, microbial genes and metabolic pathways can be detected. In contrast, amplicon sequencing only allows for the prediction of microbial genes and metabolic pathways (Durazzi et al., 2021). Shotgun sequencing integrates function, taxonomy and phylogeny and provides insights into the structure and function of the microbial community. It allows us to identify not only taxonomic units, but also genes, enzymatic reactions and metabolic pathways involved in microbial functionality. Given that there are 150 times more microbial genes than human genes, shotgun sequencing will soon enable us to understand the mechanisms behind the association of the microbiota with various diseases, including CRC (Qin et al., 2010; Wang et al., 2015).

The aim of this study was to compare the microbiome of patients with colorectal cancer and colorectal adenomas with that of generally healthy participants from different studies. With this goal in mind, we sought to conduct a real-life experiment and create a robust machine learning model that can be applied to the general population.

In a typical procedure for building a disease classifier, a certain number of individuals with and without a disease are sampled by some research group in order to obtain data for machine learning. The pool of sampled individuals is necessarily limited, by means of which their diversity is less than satisfactory. Hence, the resulting machine-learning model is necessarily overfitted to the very participants in a study. In contrast, the study in this article was conduct on as large dataset as it was possible to constellate from available sampled data from all over the world. The aim was to incorporate as rich diversity of a broad population into the resulting machine learning model. With this regard, it is reasonable to expect that at least some confounding factors are removed from the obtained disease classifier.

## 2 Methods

### 2.1 Data

Paired read sequences from 2,461 healthy participants, 380 CRC patients and 110 CRA individuals were downloaded from publicly available datasets studying different associations of different diseases and healthy controls. The main data selection criteria were the number of samples, depth of sequencing, the quality of resulting QC-ed sequences and the availability of metadata. Healthy individuals were defined as those who were reported as not having any overt disease not adverse symptoms at the time of the original study. The list of available datasets used in this study is available in Supplementary Table S1. The same dataset was used in study representing gut microbiome health index (Gupta et al., 2020). With a larger, healthy cohort, the aim was to consider the substantial variability of the human gut microbiome among healthy individuals (He et al., 2018).

### 2.2 Sequence processing

Paired-end reads were obtained from publicly available datasets using download procedures of European Nucleotide Archive^1^ (Supplementary Table S1; Supplementary material: Extended discussion) and analyzed using our custom metagenomics sequence processing pipeline MetaBakery (currently in preparation, Deutsch et al., 2022a). MetaBakery is a new implementation of the BioBakery workflow (Beghini et al., 2021) and includes tools such as KneadData v0.12.0^2^ with contaminant databases human_hg38_refMrna and hg37dec_v0.1 for quality control, MetaPhlAn 3.1.0 with database mpa_v31_CHOCOPhlAn_201901 for taxonomic analysis (for bacteria, archaea, fungi, protozoa and viruses) (Beghini et al., 2021) and HUMAnN 3.1.1 (Beghini et al., 2021) with databases full_chocophlan.v201901_v31 and uniref90_201901b_full for inferring functional genes, enzymatic reactions and metabolic pathways. In addition, MelonnPan 0.99.0 (Mallick et al., 2019) was used for the prediction of microbial metabolites. MetaBakery is containerized as a Singularity image and optimized for high performance clustering processing of large numbers of samples. For diversity assessment, Mothur 1.46.1 was integrated as part of MetaBakery pipeline utilizing biome format for diversity calculators (*n* = 35) (Schloss et al., 2009; Schloss, 2020). For this study no hand-crafted command-line parameters were used for executing the above-mentioned programs. If not instructed differently, the MetaBakery pipeline executes each program with its default parameters, as they apply to execution within the bioBakery workflow.

Minor steps of the analyses with MetaBakery were performed on a dual Xeon system with 32 CPU cores (64 hyperthreads), 512 GB RAM and 6 TB SATA hard disk at the Faculty of Electrical Engineering, University of Ljubljana, Slovenia. HPC system Vega at the Institute of Information Science^3^ and the HPC infrastructure Leo3, Leo4e of the University of Innsbruck, Austria, were utilized for heavy duty processing. In total, 980,000 CPUh were consumed.

### 2.3 Statistical analysis

Python 3.9^4^ (Van Rossum and Drake, 2009) served as the basis for our statistical analysis. We used the non-parametric Mann–Whitney test integrated in the scipy.stats library (Virtanen et al., 2020) to accurately determine the statistical significance between groups in terms of diversity and the features identified in the auto machine-learning (autoML) analysis. These features were selected by an automatic machine learning analysis based on taxonomic signatures, gene families, enzymatic reactions, metabolic pathways and predicted metabolites in the different groups (CRC, CRA, healthy). We used the Python libraries matplotlib (Hunter, 2007) and seaborn (Waskom, 2021) to visualize our results. The scikit-learn library (Pedregosa et al., 2011) in Python facilitated the linear discriminant analysis (LDA), while the preprocessing was done using the StandardScaler method. Using the LDA method, we visualized and interpreted the differences between three different clusters: CRC, CRA and healthy participants. These observations were based on taxonomic signatures, gene families, enzymatic reactions, metabolic pathways and predicted metabolites, leading to a comprehensive understanding of the data. In addition UMAP clustering was performed using JADBIO machine learning (Tsamardinos et al., 2022).

### 2.4 Automated machine learning

The web-based machine learning platform “Just Add Data Bio” (JADBIO, Ver. 1.4.105) was used to investigate potential biomarkers (Tsamardinos et al., 2022). A two-stage methodology was used for the analysis. First, the models were trained individually for each component of the data matrix, i.e., for taxonomy, functional genes, enzymatic reactions, metabolic pathways and predicted metabolites. Subsequently, an integration step was performed in which all significant features were merged, and the model was retrained. JADBIO was developed for predictive modeling and uses advanced statistical and machine learning techniques to create robust diagnostic predictive models. The analysis was systematically performed to rule out personal analytical bias and methodological statistical errors by autonomously examining different modeling settings (Deutsch and Stres, 2021; Murovec et al., 2021; Deutsch, 2022; Deutsch et al., 2022a,b). This process led to the identification of key features that allow effective discrimination between different groups. Using considerable computational resources and careful parameter tuning, JADBIO was used to model different dataset variations. The data was preprocessed to retain all rows (representing taxonomical features, gene families, enzymatic reactions and metabolic pathways) with at least 1,250 non-zero values, aiming to exclude the influence of large proportion of zeroes in the dataset. More than 2000 different model configurations were used to find the best possible model per every data matrix (Supplementary Table S2). All steps involving machine learning were used as implemented in JADBIO. Different model configurations were tested with different preprocessing steps, feature selectors, feature selection hyperparamters, predictive algorithms and hyperparameters were tested (Supplementary Table S2; Supplementary material: Extended discussion). The analysis included features extracted from samples of different projects and groups, with the data split 70:30 into training and test datasets. The training dataset was used to develop the model, while the test dataset evaluated its performance (Deutsch and Stres, 2021; Murovec et al., 2021; Deutsch, 2022; Deutsch et al., 2022a,b). Receiver operating characteristic curves (ROC) were generated for all groups studied to evaluate the model. These curves graphically represented the trade-off between the rate of true-positive findings (sensitivity) and the rate of false-positive findings (1-specificity). Individual conditional expectation plots (ICE) were used for depth to illustrate the differential contribution of each feature to the predictive power of the model. Progressive feature inclusion plots were also created to provide insight into the impact of feature inclusion on model performance.

## 3 Results

### 3.1 Diversity

**Figure 1:**
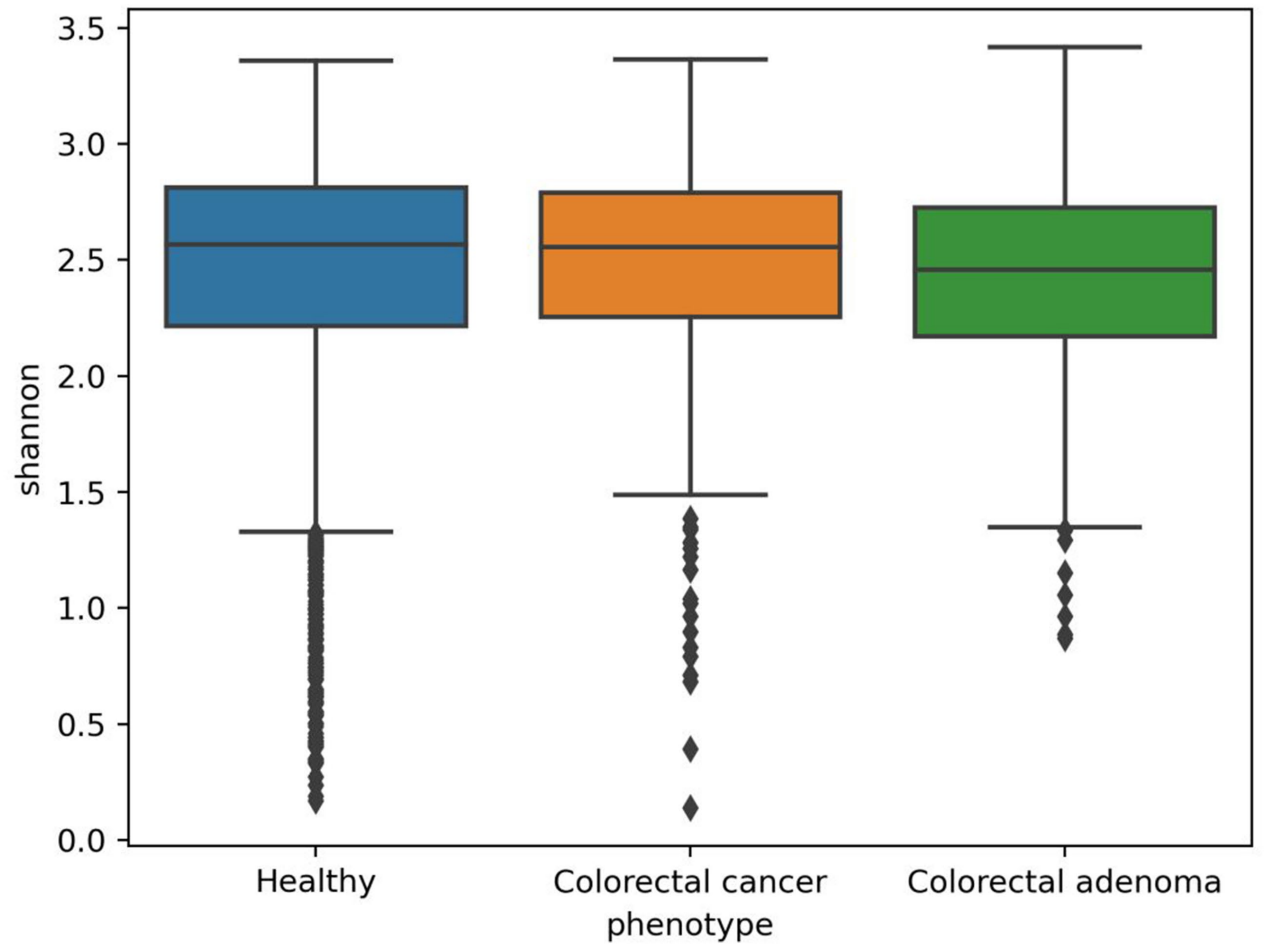
Boxplots representing Shannon diversity metrics for healthy individuals and patients with colorectal cancer or colorectal adenoma.

The in-house analytical pipeline MetaBakery (in preparation, Deutsch et al., 2022a) was used to preprocess the sequence data with integrated tool KneadData^5^ and to analyze the sequences at the level of taxonomy [MetaPhlAn3 (Beghini et al., 2021)], diversity [Mothur (Schloss et al., 2009)], functional genes, enzymatic reactions and metabolic pathways [HUMAnN3 (Beghini et al., 2021)] and predicted metabolites [MelonnPan (Mallick et al., 2019)]. Sequences from 2,461 healthy individuals, 380 CRC patients and 110 individuals with confirmed CRA were used for the analysis. A total of 1839 taxonomic units (kingdoms, phyla, clades, orders, families, genera and species) including archaea, bacteria, protozoa and viruses, 80,372 gene families, 34,008 enzymatic reactions, 31,555 metabolic pathways and 81 predicted metabolites were identified and analyzed in the human gut microbiota. 19 different diversity metrics were used to compare all three groups and determine the presence of differences. Although in most cases the diversity metrics were higher in the CRC and CRA groups, these differences were not significant, including the Shannon diversity index (Figure 1) as determined by the Mann–Whitney test (Supplementary Table S3; Supplementary Figure S1).

### 3.2 LDA analysis

**Figure 2:**
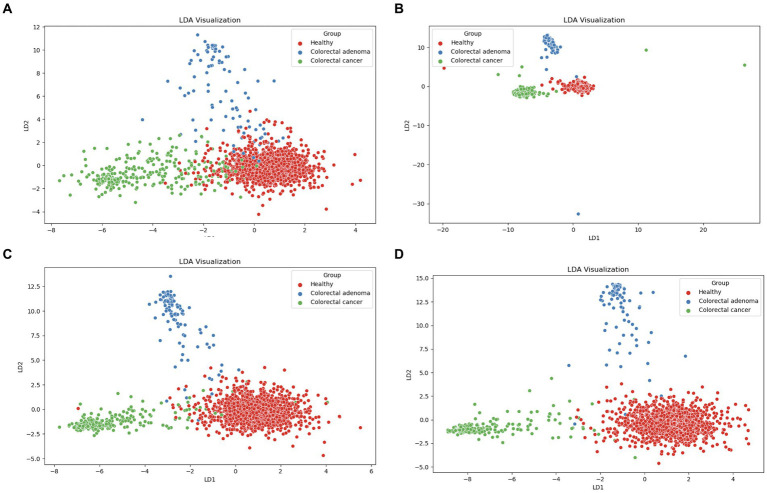
LDA scores plots of components one and two for healthy (red), patients with CRC (green) and CRA (blue): **(A)** taxonomy, **(B)** gene families, **(C)** enzymatic reactions, and **(D)** metabolic pathways.

Using the scikit-learn Python library, linear discriminant analysis (LDA) was used to explore potential differences between healthy individuals, CRA and CRC patients in the five data matrices (taxonomy, functional genes, enzymatic reactions, metabolic pathways and predicted metabolites). As shown in Figure 2, LDA clustering effectively discriminates between CRC, CRA and healthy individuals based on four different metagenomic fingerprints (taxonomy in Figure 2A, functional genes in Figure 2B, enzymatic reactions in Figure 2C and metabolic pathways in Figure 2D). However, no clear LDA cluster separation was observed for the predicted metabolites (Supplementary Figure S2). In addition, UMAP analysis was performed using JADBIO (Supplementary Figure S3).

### 3.3 Machine learning results

Although clear separation was observed in only four datasets (taxonomy, genes, enzymatic reactions and metabolic pathways), all five metagenomics data matrices (taxonomy data, functional genes, enzymatic reactions, metabolic pathways and predicted metabolites) were used for automatic machine learning using the JADBIO web-based tool. All matrices were prepared such that rows with at least 1,250 non-zero entries were retained in the dataset.

**Figure 3:**
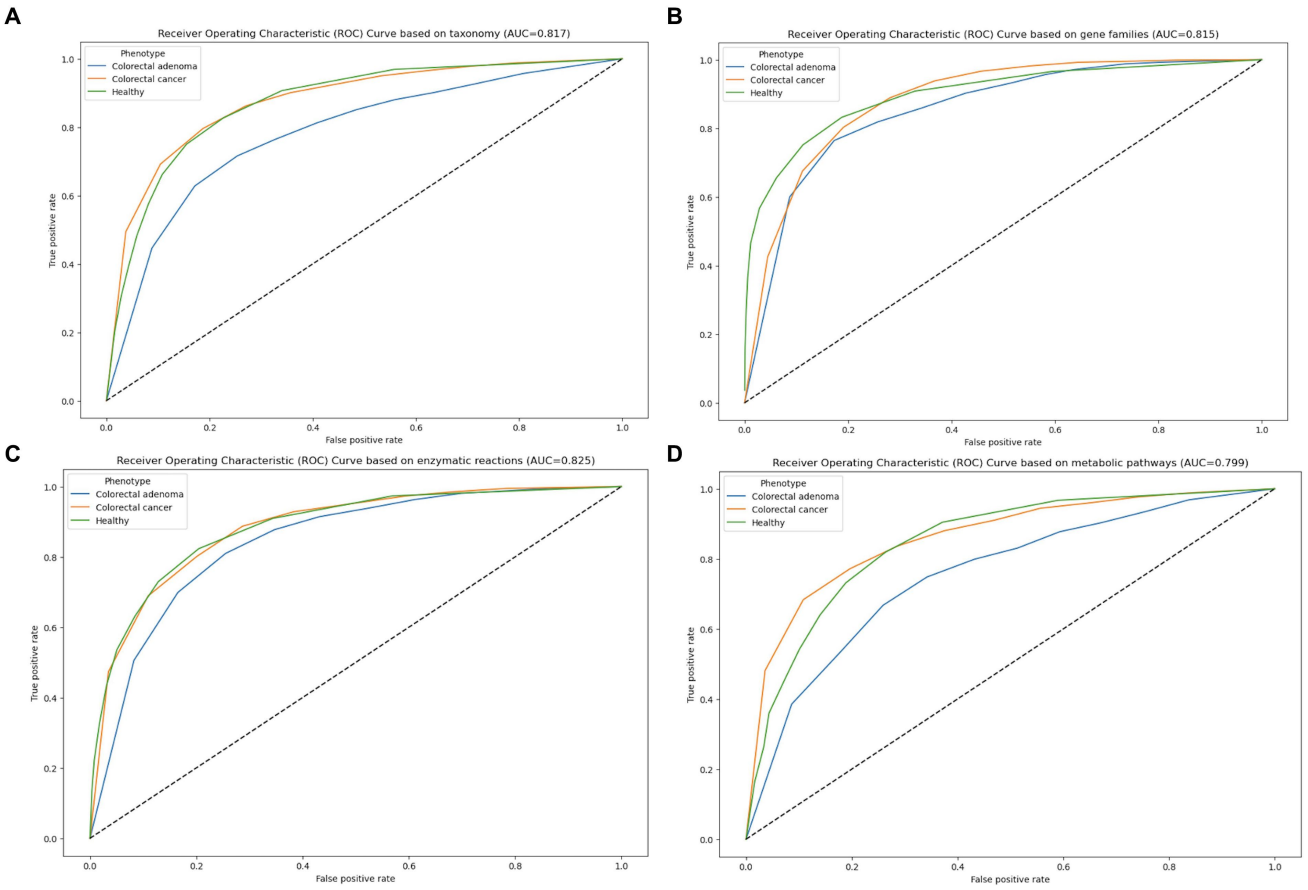
ROC plots for classification between healthy individuals (green), CRC (orange) and CRA (blue) patients based on taxonomy **(A)**, functional genes **(B)**, enzymatic reactions, **(C)** and metabolic pathways **(D)**.

Based on the 1839 categories describing the taxonomic data of four different kingdoms (Archaea, Bacteria, Protozoa and Viruses), the models were trained using extensive tuning effort in search of biologically meaningful distinguishing features between all three groups. All important features were representative of the Bacteria kingdom and the best performing model was Classification Random Forest training 1,000 trees with deviance splitting criterion, minimum leaf size = 2, splits = 1, alpha = 1 and variables to split = 1.0 sqrt (nvars) according to JADBIO, after testing more than 2000 different configurations. More than 25 features were selected as the most appropriate to achieve the best possible differentiation between all three groups (AUC = 0.817), but the first ten taxonomic units can achieve more than 95% successful performance for differentiation (Figure 3A; Supplementary Figure S4; Supplementary Table S4). This model was tested with all 25 selected features using test data and achieved a performance of AUC = 0.787.

HUMAnN3 (Beghini et al., 2021), integrated in our MetaBakery pipeline, was used to assess the functional potential of the microbiome. Functional genes were determined using the UniRef database (Suzek et al., 2007, 2015). 80.372 functional genes were discovered in the samples and 70% of the total dataset was used to find the best possible model. The best possible model was Classification Random Forest training 1,000 trees with deviance splitting criterion, minimum leaf size = 3, splits = 1, alpha = 1 and variables to split = 0.577 sqrt (nvars) with an area under the curve value of 0.815 (Figure 3B). From the entire pool of genes, 25 of them were selected as the most important features for differentiation. However, a classification performance of 100% was achieved with the first 15 of them (Supplementary Figure S5). The model was tested on 30% of the entire dataset and achieved an accuracy of AUC = 0.822.

The aggregation of functional gene information into enzymatic reactions (Figure 3C) led us to model 34,008 enzymatic reactions. The best model was Classification Random Forest training 1,000 trees with deviance splitting criterion, minimum leaf size = 1, splits = 1, alpha = 1 and variables to split = 0.577 sqrt (nvars), with an Area under the Curve (AUC) value of 0.825. 25 different features were identified as the most important for discrimination and the first 18 of them can achieve a prediction performance of 100% (Supplementary Figure S5; Supplementary Table S4). The model was tested and achieved a performance with an AUC value of 0.812.

The aggregation of enzymatic reactions into metabolic pathways (Figure 3D) led to the modeling of 31,555 metabolic pathways. The best model was Classification Random Forest training 100 trees with deviance splitting criterion, minimum leaf size = 2, splits = 1, alpha = 1 and variables to split = 0.577 sqrt (nvars), with an area under the curve (AUC) value of 0.799.25 different features were identified as the most important for discrimination and the first 13 of them can reach a prediction performance of 100% (Supplementary Figure S6; Supplementary Table S4). The model was tested on the test dataset and achieved a performance with an AUC value of 0.768.

The LDA analysis and clustering visualizations have already shown that the lowest expected performance can be obtained when modeling the predicted metabolite data obtained with the MelonnPan tool (Mallick et al., 2019). This was also confirmed with Classification Random Forest training 1,000 trees with deviance splitting criterion, minimum leaf size = 2, splits = 1, alpha = 1 and variables to split = 1.0 sqrt (nvars) as the best prediction algorithm based on 81 predicted metabolites. However, the performance of this model was low (AUC = 0.621). The performance on the test dataset was even lower (AUC = 0.606) (Supplementary Figures S7, S8; Supplementary Table S4).

All features identified by JADBIO through automatic machine learning were also tested using the Mann–Whitney statistics to check correctness and significance between groups for each feature. Most comparisons for each feature in the areas of taxonomy, functional genes, enzymatic reactions, and metabolic pathways were statistically significant, especially when comparing CRC and healthy controls. Comparisons of CRA and healthy controls on the one hand or CRC and CRA on the other were less significant. The differences in the selected predicted metabolites were not significant (Supplementary Table S5).

In the final step of the machine learning analysis, the most important features were integrated into a data set and the machine learning was repeated on this reduced data set. Classification Random Forest trained 1,000 trees with deviance splitting criterion, minimum leaf size = 3, splits = 1, alpha = 1 and variables to split 0.816 sqrt was selected as the most successful for aggressive feature selection and 25 out of 120 features were selected as the most important for classification (5 belong to taxonomy–kingdom bacteria, 12 to gene families, 5 to enzymatic reactions and 3 to metabolic pathways). None of the predicted metabolites from the first step were selected in the second step. The final performance of this model was 0.87 (AUC).

## 4 Discussion

A total of 2,951 stool samples from different studies, including healthy individuals as well as those with CRC and CRA, were subjected to comparative analysis. Our MetaBakery pipeline was used for sequence processing. Comprehensive data matrices were used that included various features such as microbial taxonomy (1839 taxonomic units), functional genes (80,372 genes), enzymatic reactions (34,008 enzymes), metabolic pathways (31,555 metabolic pathways), and predicted metabolites (81 metabolites). In addition, we integrated 19 different diversity matrices calculated using methods consistent with Mothur’s approach.

We showed that there is no statistically significant difference in microbial diversity in patients with colorectal cancer (CRC). These results are consistent with some other studies suggesting that microbial diversity and richness may increase in colorectal cancer patients (Feng et al., 2015; Thomas et al., 2019; Qi et al., 2022; Liu J. et al., 2023). To further investigate possible differences, we first performed a comprehensive analysis of the entire dataset using linear discriminant analysis (LDA) to identify possible clusters. Significant differences emerged in four different metagenomic data matrices (taxonomy, functional genes, enzymatic reactions and metabolic pathways), which formed separate clusters for each group (healthy, CRC, CRA). A clear difference was seen between the healthy and CRC patient groups. However, the CRA patients were consistently positioned between the healthy controls and the CRC patients, emphasizing that CRA represents a closer step to the development of CRC in terms of the composition of the microbiome. CRA is considered as a stage 0 in development of intramucosal carcinoma and can progress into malignant forms, which is also known as an adenoma-carcinoma sequence. The most important question here is whether the change in the microbiome is the consequence of the development of the disease or whether the disease is a consequence of the change in the microbiome. Given the obvious differences observed in LDA analysis between healthy microbiomes, CRC and CRA samples, machine learning (ML) analysis was performed. Datasets from different studies were used to represent real-world scenarios and achieve a level of variability that corresponds to natural conditions rather than exerting excessive control.

We obtained consistent model performance with AUC values around 0.8 for all data inputs. In this study, we present several groups of microbial taxa, functional genes, enzymatic reactions and metabolic pathways that offer potential for the prediagnostic evaluation of CRC and CRA that represent an early stage in the development of CRC. Several CRC biomarker species were independently identified in the different studies by univariate statistics (Segata et al., 2011): *Fusobacterium nucleatum*, *Solobacterium moorei*, *Porphyromonas asaccharolytica*, *Parvimonas micra*, *Peptostreptococcus stomatis* and *Parvimonas* ssp. (Kostic et al., 2012, 2013; Thomas et al., 2019; Mizutani et al., 2020; Qi et al., 2022). In our study different groups of taxa, from phylum to genera, were identified important for distinguishing between different conditions (health, CRC or CRA). Many previous studies focused exclusively on a binary classification including only colorectal cancers and healthy individuals, which may have introduced bias. The detection of individuals with CRA, a precursor of CRC, is important from a diagnostic point of view.

In recent years, research into the functionality of the microbiome has become increasingly important. The emergence of microbial metagenomics has highlighted that data modeling must also be approached from the perspective of microbial functionality, as the ratio of human to microbial genes is 1:150 (Qin et al., 2010). This shift is crucial as it provides a better understanding of overall microbial functionality rather than microbial taxonomy (Deschênes et al., 2023). Furthermore, it promises to reveal why certain components of the microbiome may be associated with the occurrence of various diseases. With this in mind, our investigations extend to microbial functional potential, which includes functional genes, enzymatic reactions, metabolic pathways and predicted metabolites.

Our initial focus on functional genes, enzymatic reactions and metabolic pathways has led to promising results and moderate classification accuracy. Based on the UniRef database (Suzek et al., 2007, 2015), 15 different gene families were discovered that are important for classification between all three groups. Most of the discovered gene families belong to the human gut microbiota. Moreover, for example, the gene family A0A015S3B6|unclassified belongs to the protein of *Bacteroides fragilis*, which has also been previously mentioned as one of the biomarker candidates for CRC (Pandey et al., 2023). The gene family A0A078RCV9 belongs to *Phocaeicola vulgatus*, (formerly *Bacteroides vulgatus*, which was already associated with CRC in 1995) (Moore and Moore, 1995; Lucas et al., 2017; Vu et al., 2022). The gene families A0A174XNP7 (belonging to *Flavonifractor plautii*) and A0A174Q9G9 (*Bacteroides intestinalis*) have been associated with colorectal cancer patients in India (Gupta et al., 2019).

The most important enzymatic reaction is 3.5.1.88-RXN according to feature selection, which belongs to *Holdemanella biformis*, one of the species that can act anti-oncogenically through the production of SCFAs (Zagato et al., 2020). Reaction 3.4.21.92-RXN belongs to *Lawsonibacter asaccharolyticus*, previously associated with acetate, a potential therapeutic agent in the treatment of colorectal cancer (Marques et al., 2013; Sahuri-Arisoylu et al., 2021; Dong et al., 2023). Reatcion 3.2.1.1-RXN belongs to *Clostridium* sp. *CAG_58*, the most important taxon from the taxonomic data feature selection, was previously associated with adiposity. Higher obesity has generally been associated with an increased likelihood of CRC (Bull et al., 2020; Asnicar et al., 2021). Reaction 2.5.1.64-RXN belongs to *Klebsiella oxytoca*, another microbial species that has been isolated from patients with CRC and is one of the reasons for the increased inflammation in these patients due to biofilm formation (Abbas et al., 2020). One of the most interesting features discovered in the enzymatic reactions was 2.3.1.180-RXN belonging to *Fusobacterium nucleatum*, which, as mentioned above, was one of the most important species-level biomarkers observed in other studies (Kostic et al., 2012, 2013). Even though we did not observe this species at the taxonomic level, we did observe this reaction. Reaction 2PGADEHYDRAT-RXN was also identified and belongs to *Collinsella aerofaciens*, a microbe observed in the stool of patients with elevated blood levels (Chénard et al., 2020).

MetaCyc (Caspi et al., 2020) metabolic pathways were also identified as important features for classification. The most important feature in this regard was ARO-PWY: chorismate biosynthesis I. Chorismate is also a precursor of tryptophan. It was observed that the reduction in the amount of tryptophan is proportional to the poor quality of life of colorectal cancer patients (Zhang et al., 2019). The next metabolic pathway was ARGSYN-PWY: L-arginine biosynthesis I. It was observed that supplementation with L-arginine can alleviate intestinal inflammation. Increased intestinal inflammation was observed to be associated with the initiation and progression of CRC (Zhang et al., 2021; Liu Y. et al., 2023). Arginine was also observed to have significant diagnostic value for CRC patients (Yi et al., 2023).

However, the AUC values for the predicted metabolites were lower compared to other data matrices. Pantothenate was observed to be the most important feature. Pantothenate was previously observed as an important metabolite for the diagnosis of CRC patients (Yi et al., 2023). Putrescine, the second most important feature, is a polyamine that is basically involved in all steps of tumorigenesis (Sánchez-Alcoholado et al., 2021).

Although there are still no definitive explanations for many discovered genes, enzymes and metabolic pathways, this uncertainty will decrease over time. For example, it is expected that questions about the significance of a particular metabolic pathway for the classification of a particular disease will be clarified. We have also ventured into the prediction of metabolites using relaxation networks such as those included in MelonnPan. Although the results were statistically insignificant, it is plausible that subsequent iterations of this tool or similar tools could improve the prediction of metabolites. This potential breakthrough could facilitate the linking of metabolite predictions with results from fecal or blood metabolome analyses (Šket et al., 2020; Deutsch et al., 2022a). Such an integrated approach could reveal new dimensions in the understanding of microbe-host relationships, enriching our knowledge and potentially paving the way for practical clinical applications. With the approach outlined in this study, we have shown that it is possible to develop robust prediagnostic methods for colorectal cancer detection based on microbial fingerprints (Cammarota et al., 2020; Su et al., 2022; Zhou et al., 2024) integrating all layers of information (taxonomy, diversity, functional genes, enzymatic reactions, metabolic pathways, metabolites). One of the limitations mirroring the current status of the research in this fields and of our study is the lack of external clinical datasets of sufficient high quality of sequences and metadata to validate our models. However, with the advent of novel datasets the models created in this study could be used in larger studies in the future to evaluate the results obtained. Nevertheless, the research presented here provides one of the first important steps toward efficient, reproducible and tractable classification of CRC and CRA samples in a form of prediagnostic informative tool.

## Data Availability

The datasets presented in this study can be found in online repositories. The names of the repository/repositories and accession number(s) can be found in the article/Supplementary material.

## Footnotes

^1^ https://ena-docs.readthedocs.io/en/latest/retrieval/file-download.html

^2^https://huttenhower.sph.harvard.edu/kneaddata/, accessed October 10, 2023.

^3^ www.izum.si

^4^https://www.python.org/, accessed October 10, 2023.

^5^https://huttenhower.sph.harvard.edu/kneaddata/, accessed October 10, 2023.

## Supplementary material

SUPPLEMENTARY TABLE S1 Accession numbers of used studies.

SUPPLEMENTARY TABLE S2 The list of tested model configurations.

SUPPLEMENTARY TABLE S3 Mann–Whitney statistic of diversity metrics.

SUPPLEMENTARY TABLE S4 Important features identified by JADBIO Auto-ML approach.

SUPPLEMENTARY TABLE S5 Mann–Whitney statistics of important features, extended discussion—limitations of ML approaches in microbiome studies, extended discussion—rationale of using paired read sequences.
