# Supplementary material for: Predictive modeling of colorectal cancer using exhaustive analysis of microbiome information layers available from public metagenomic data
Source: Front Microbiol. 2024 Aug 26;15:1426407. doi: 10.3389/fmicb.2024.1426407 (PMC11381387; doi:10.3389/fmicb.2024.1426407)
Supplement: Supplementary file 1 [file Data_Sheet_1.docx]

Supplementary Material

Predictive modeling of colorectal cancer using metagenomic data: A real-life experiment

Boštjan Murovec^1^, Leon Deutsch^2,3^, Blaž Stres^2,4,5,6*^

^1^Faculty of Electrical Engineering, University of Ljubljana, Ljubljana, Slovenia

^2^Department of Animal Science, Biotechnical faculty, University of Ljubljana, Ljubljana, Slovenia

^3^The NU, The NU B.V., Leiden, The Netherlands

^4^D13 Department of Catalysis and Chemical reaction Engineering, National Institute of Chemistry, Ljubljana, Slovenia

^5^Institute of Sanitary Engineering, Faculty of Civil and Geodetic Engineering, Ljubljana, Slovenia

^6^Department of Automation, Biocybernetics and Robotics, Jožef Stefan Institute, Ljubljana, Slovenia

Extended discussion

**Limitations of ML approaches in microbiome studies**

Machine learning (ML) approaches in microbiome studies have several limitations, primarily related to the complexities of accounting for confounding factors and the need for improved feature selection, nonlinear modeling capabilities, robust model evaluation, scalable analyses, and integrative modeling. While ML models can incorporate metadata and other contextual information to learn complex relationships and predict outcomes more accurately, they often struggle with the high-dimensional, multivariate nature of microbiome data. Additionally, although ML techniques can automate and scale analyses, identify relevant microbial features, and integrate multiple data modalities, ensuring rigorous cross-validation and addressing potential biases and overfitting remain critical challenges in translating microbiome research findings into clinical applications (Hernández Medina et al., 2022; D’Elia et al., 2023; Papoutsoglou et al., 2023; Kumar et al., 2024).

1. Accounting for Confounding Factors:

- ML models can be designed to explicitly incorporate metadata and other contextual information, such as host characteristics, environmental factors, and clinical variables, as part of the input features.

- This allows the models to learn the complex relationships between the microbiome composition and the confounding factors, enabling better prediction of outcomes while accounting for these confounding effects.

2. Improved Feature Selection:

- ML techniques, such as regularized regression (e.g., Lasso, Elastic Net) and tree-based methods (e.g., Random Forests), can automatically identify the most informative microbial features (taxa, genes, or functions) that are predictive of the outcome of interest, while accounting for the effects of confounding factors.

- This feature selection process helps to focus the analysis on the most relevant microbiome signatures, reducing the impact of irrelevant or redundant features that may be influenced by confounding factors.

3. Nonlinear Modeling Capabilities:

- Many ML algorithms, such as neural networks and gradient boosting, can capture complex nonlinear relationships between the microbiome and the outcome of interest, which may be obscured by traditional statistical methods.

- This ability to model nonlinear effects can help uncover microbiome-outcome associations that may be missed by linear models, which can be particularly important when dealing with the complex, multivariate nature of microbiome data.

4. Robust Model Evaluation:

- ML models can be evaluated using rigorous cross-validation techniques, which help to assess the model's performance on unseen data and ensure that the results are not biased by confounding factors or overfitting.

- This robust model evaluation approach provides a more realistic estimate of the model's predictive power, which is crucial for translating microbiome research findings into clinical applications.

5. Automated and Scalable Analyses:

- ML-based workflows can be automated and scaled to handle large, high-dimensional microbiome datasets, which is particularly important when dealing with the growing volume of microbiome data generated by various studies.

- This automation and scalability can help researchers efficiently explore the effects of confounding factors and identify robust microbiome-based biomarkers, without being limited by manual, time-consuming data analysis processes.

6. Integrative Modeling:

- ML techniques can be used to integrate multiple data modalities, such as microbiome, host genetics, clinical metadata, and environmental factors, to build more comprehensive and accurate predictive models.

- This integrative approach can help disentangle the complex interplay between the microbiome and various confounding factors, leading to a better understanding of the underlying mechanisms and more reliable predictions.

**Rationale of using paired read sequences**

There are several key reasons supporting our decision to focus solely on paired-end read sequences:

1. Improved Taxonomic Resolution:

- Paired-end sequencing allows for the full length of the targeted amplicon (e.g., 16S rRNA gene) to be sequenced, providing more complete and accurate taxonomic information compared to single-end sequencing.

- The additional sequence information from paired-end reads enables better taxonomic classification, especially at the species or strain level, which is crucial for understanding the functional and ecological roles of microbes in the community.

2. Reduced Bias and Errors:

- Relying solely on single-end reads can introduce biases and errors, as the partial sequence information may not be sufficient for accurate taxonomic assignment, particularly for closely related taxa.

- Paired-end sequencing helps to mitigate these issues by providing more complete sequence data, leading to more reliable taxonomic profiling and community composition analysis.

3. Consistency Across Studies:

- Focusing on paired-end read sequences enables better standardization and comparability of microbiome data across different studies, as the same amplicon region can be targeted and analyzed consistently.

- This consistency is important for meta-analyses, where data from multiple studies need to be integrated and compared to draw robust conclusions about microbial community patterns and dynamics.

4. Handling Sequence Overlaps:

- In paired-end sequencing, the forward and reverse reads often overlap, providing an opportunity to merge the overlapping regions and generate a single, higher-quality consensus sequence.

- Merging overlapping reads can help to improve sequence quality, remove potential sequencing errors, and provide a more accurate representation of the original DNA fragment.

5. Increased Statistical Power:

- By focusing on paired-end read sequences, the meta-analysis can leverage the increased statistical power provided by the additional sequence information, leading to more robust and reliable conclusions about microbial community patterns and associations.

6. Addressing Limitations of Single-End Sequencing:

- Single-end sequencing can suffer from limitations, such as reduced taxonomic resolution, increased risk of chimeric sequence formation, and difficulties in handling sequence ambiguities.

- Focusing on paired-end read sequences helps to overcome these limitations and provides a more comprehensive and accurate representation of the microbial community.

In summary, the emphasis on paired-end read sequences in microbiome meta-analyses is crucial for improving taxonomic resolution, reducing biases and errors, ensuring consistency across studies, handling sequence overlaps, increasing statistical power, and addressing the limitations of single-end sequencing approaches. This approach ultimately leads to more robust and reliable conclusions about the structure and dynamics of microbial communities (Pinna et al., 2019; Wasimuddin et al., 2020; Dacey and Chain, 2021; Borgman et al., 2022).

**References**

Borgman, J., Stark, K., Carson, J., and Hauser, L. (2022). Deep Learning Encoding for Rapid Sequence Identification on Microbiome Data. *Frontiers in Bioinformatics* 2, 871256. doi: 10.3389/FBINF.2022.871256/BIBTEX

Dacey, D. P., and Chain, F. J. J. (2021). Concatenation of paired-end reads improves taxonomic classification of amplicons for profiling microbial communities. *BMC Bioinformatics* 22. doi: 10.1186/S12859-021-04410-2

D’Elia, D., Truu, J., Lahti, L., Berland, M., Papoutsoglou, G., Ceci, M., et al. (2023). Advancing microbiome research with machine learning: key findings from the ML4Microbiome COST action. *Front Microbiol* 14. doi: 10.3389/FMICB.2023.1257002

Hernández Medina, R., Kutuzova, S., Nielsen, K. N., Johansen, J., Hansen, L. H., Nielsen, M., et al. (2022). Machine learning and deep learning applications in microbiome research. *ISME Communications 2022 2:1* 2, 1–7. doi: 10.1038/s43705-022-00182-9

Kumar, B., Lorusso, E., Fosso, B., and Pesole, G. (2024). A comprehensive overview of microbiome data in the light of machine learning applications: categorization, accessibility, and future directions. *Front Microbiol* 15, 1343572. doi: 10.3389/FMICB.2024.1343572/BIBTEX

Papoutsoglou, G., Tarazona, S., Lopes, M. B., Klammsteiner, T., Ibrahimi, E., Eckenberger, J., et al. (2023). Machine learning approaches in microbiome research: challenges and best practices. *Front Microbiol* 14. doi: 10.3389/FMICB.2023.1261889

Pinna, N. K., Dutta, A., Haque, M. M., and Mande, S. S. (2019). Can targeting non-contiguous V-regions with paired-end sequencing improve 16S rRNA-based taxonomic resolution of microbiomes?: An in silico evaluation. *Front Genet* 10, 437346. doi: 10.3389/FGENE.2019.00653/BIBTEX

Wasimuddin, Schlaeppi, K., Ronchi, F., Leib, S. L., Erb, M., and Ramette, A. (2020). Evaluation of primer pairs for microbiome profiling from soils to humans within the One Health framework. *Mol Ecol Resour* 20, 1558. doi: 10.1111/1755-0998.13215


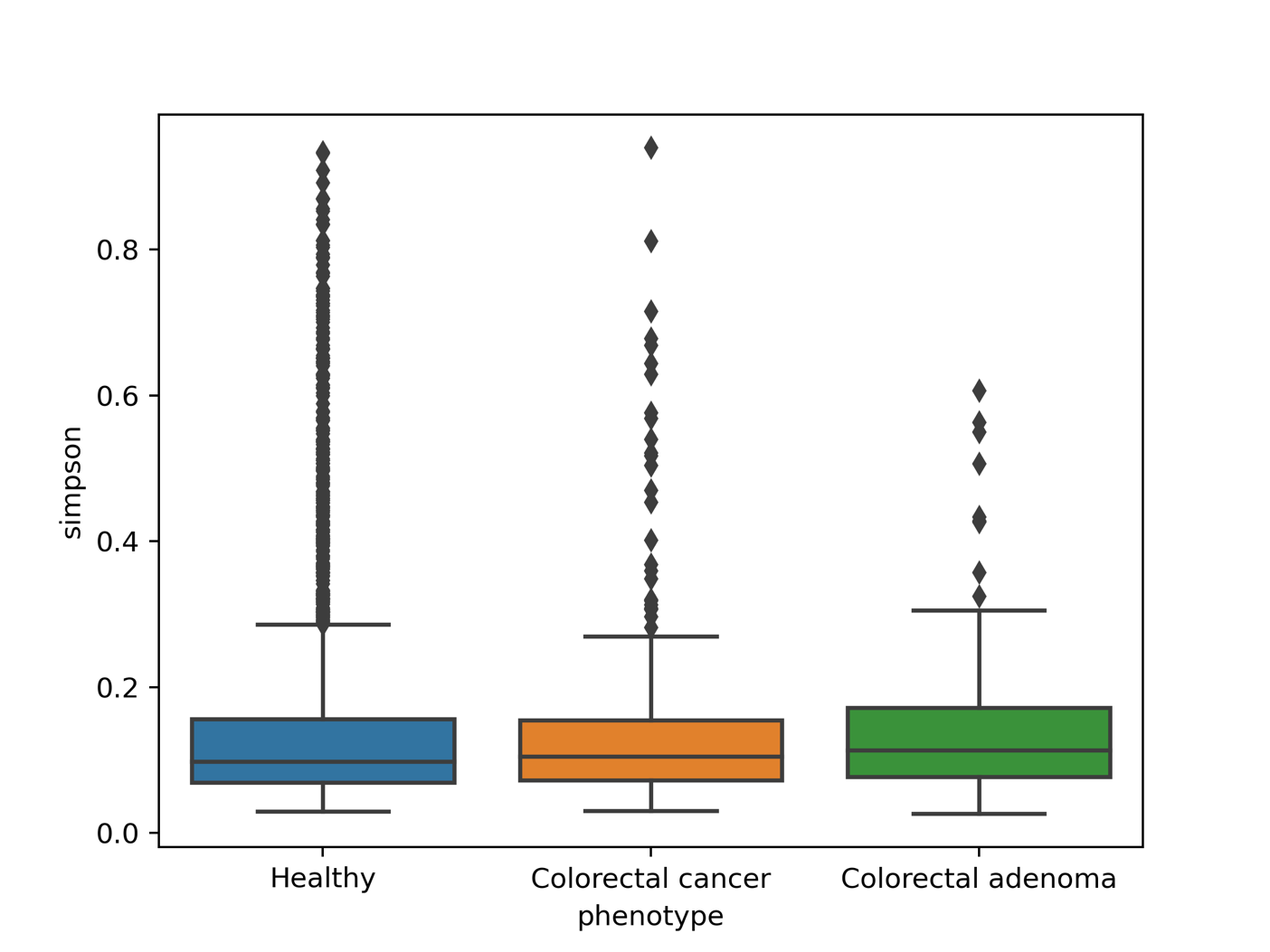


**Supplementary Figure 1.** Boxplot representing Simpson diversity metric for healthy individuals and patients with colorectal cancer or colorectal adenoma.


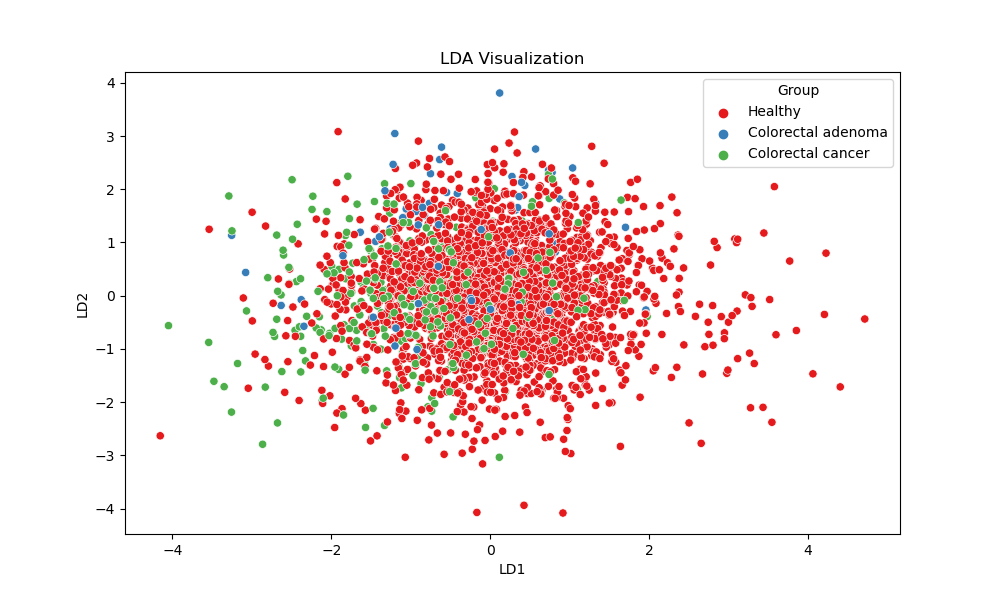


**Supplementary Figure 2.** LDA scores plots of components one and two for healthy individuals (red), CRC patients (green) and CRA patients (blue) for predicted metabolites with MelonnPan.


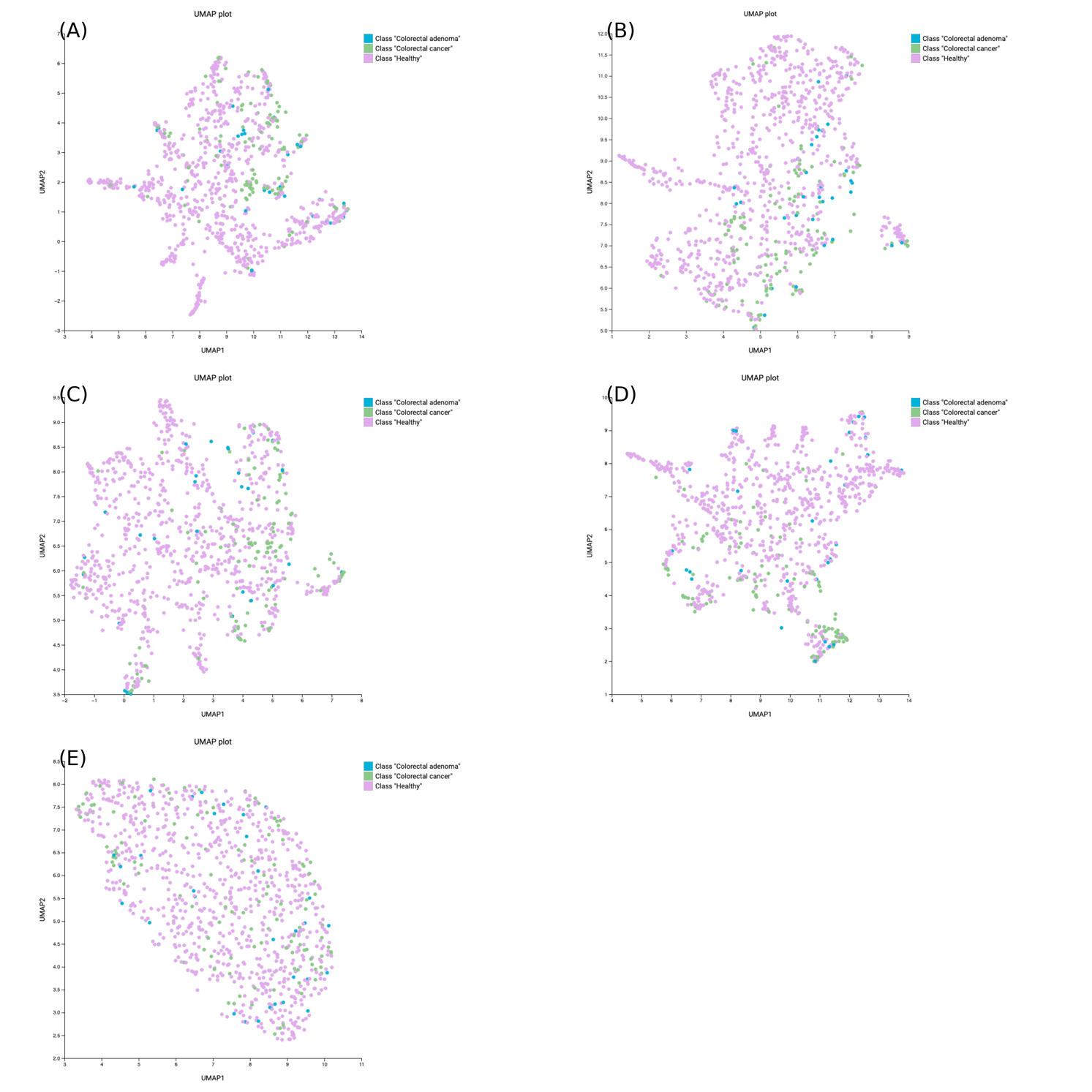


**Supplementary Figure 3.** UMAP clustering of testing data for colorectal adenoma (blue), colorectal cancer (green) and healthy individuals (pink).


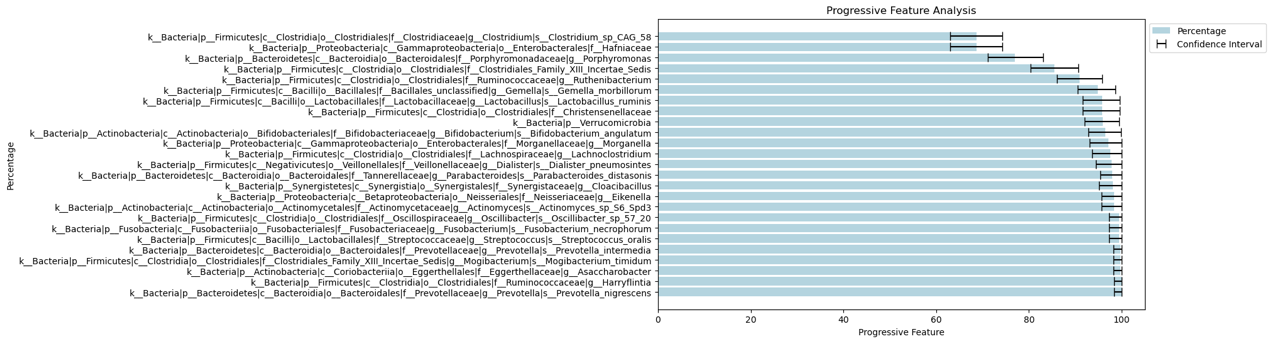


**Supplementary Figure 4.** ICE plot for 25 taxonomical features important for classification between healthy individual, CRC and CRA patients.


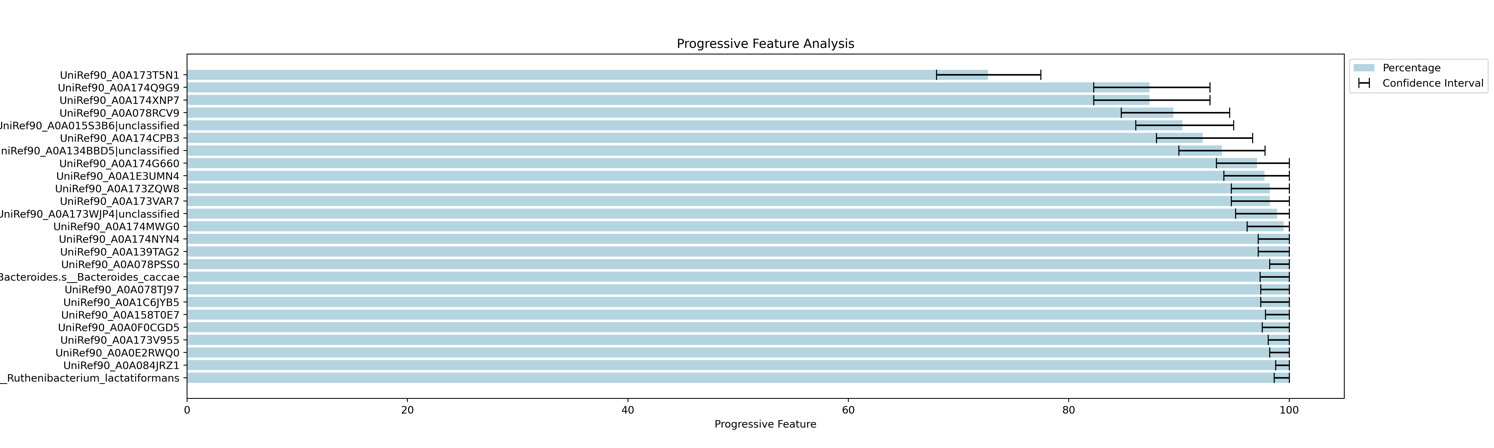


**Supplementary Figure 5.** ICE plot for 25 functional genes important for classification between healthy individual, CRC and CRA patients.


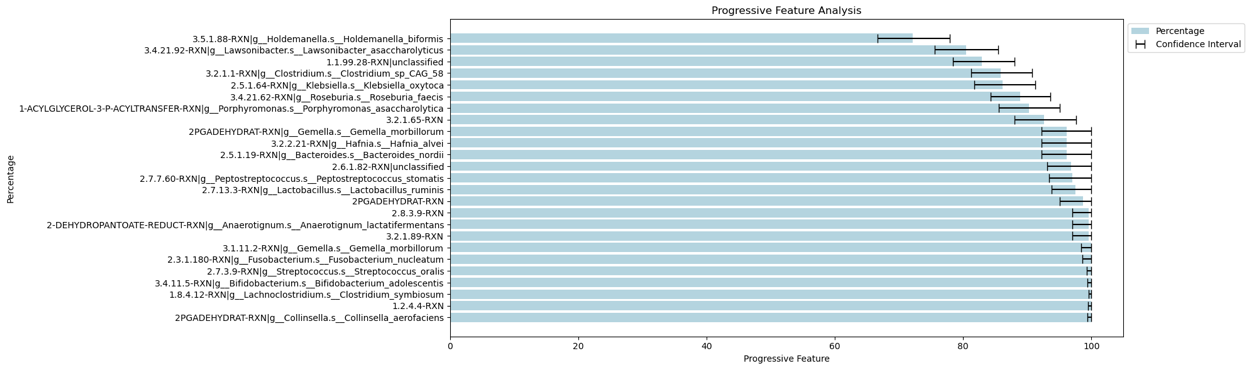


**Supplementary Figure 6.** ICE plot for 25 enzymatic reactions important for classification between healthy individual, CRC and CRA patients.


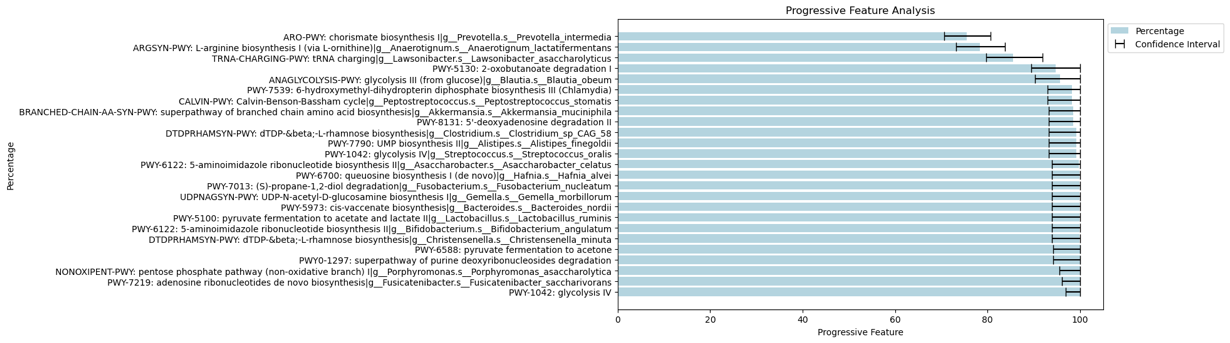


**Supplementary Figure 7.** ICE plot for 25 metabolic pathways important for classification between healthy individual, CRC and CRA patients.


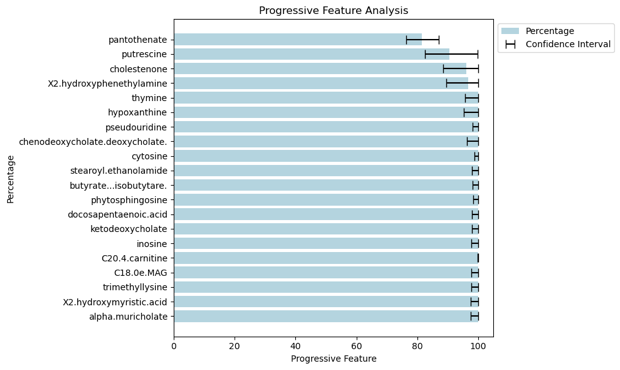


**Supplementary Figure 8.** ICE plot for 25 predicted metabolites important for classification between healthy individual, CRC and CRA patients.


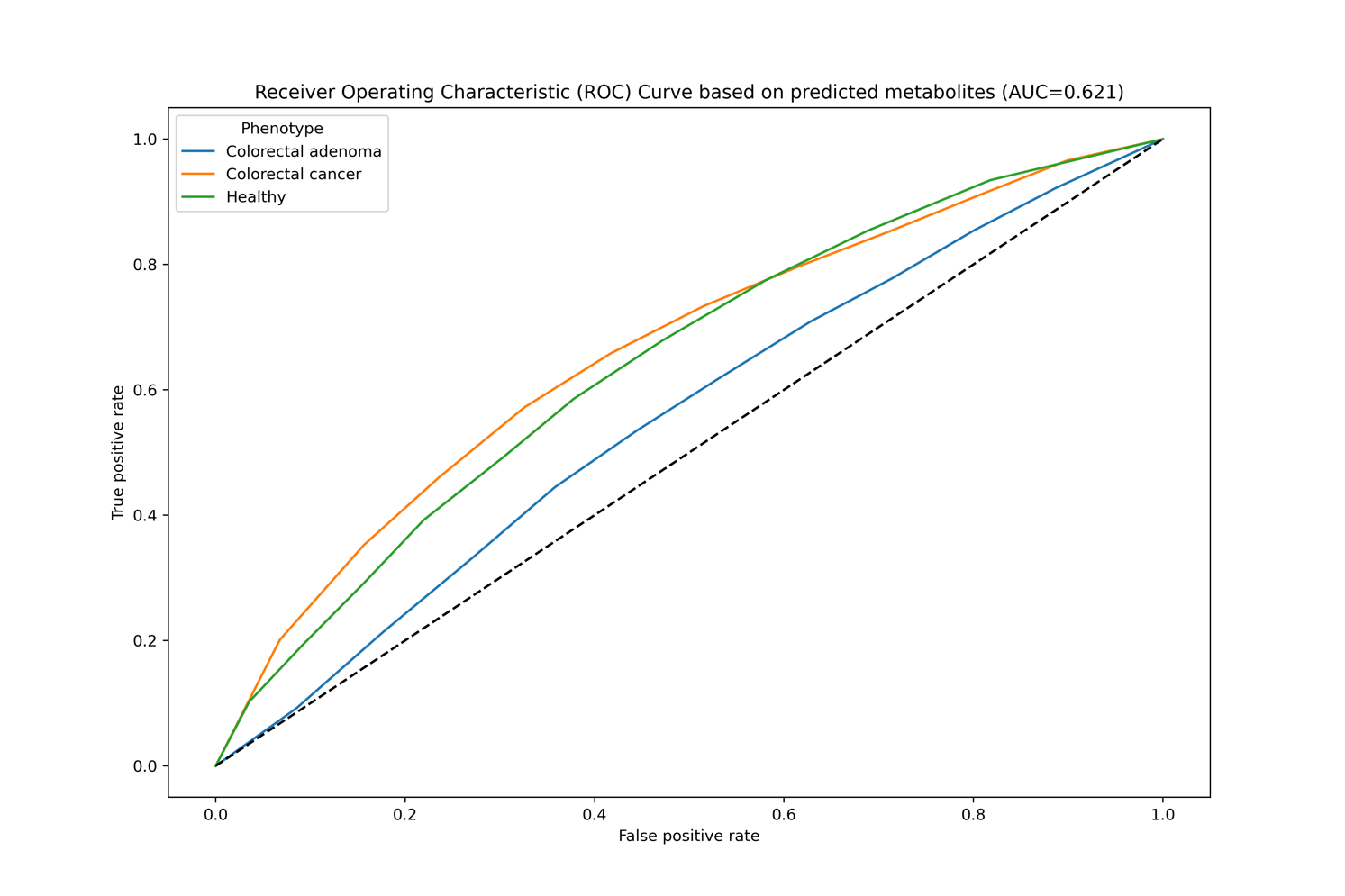


**Supplementary Figure 9.** ROC plot for 25 predicted metabolites important for classification between healthy individuals (green), CRC (orange) and CRA (blue) patients.
